# Cervical cancer in Northern Tanzania—What do women living with HIV know

**DOI:** 10.3389/fonc.2022.957325

**Published:** 2023-01-09

**Authors:** Dorah Mrema, James Samwel Ngocho, Alex Mremi, Maryam Amour, Rogathe Machange, Benjamin C. Shayo, Julius P. Alloyce, Evaline Ndosi, Beatus T. Shirima, Device Fande, Rahma Shehoza, Emmanuel Balandya, Bruno Sunguya, Stephen E. Mshana, Alfred K. Mteta, Eligius Lyamuya, John Bartlett, Blandina T. Mmbaga

**Affiliations:** ^1^ Kilimanjaro Christian Medical University College, Moshi, Kilimanjaro, Tanzania; ^2^ Department of Obstetrics and Gynaecology, Kilimanjaro Christian Medical Centre, Moshi, Tanzania; ^3^ Kilimanjaro Clinical Research Institute, Moshi, Kilimanjaro, Tanzania; ^4^ Muhimbili University of Health and Allied Sciences, Dar-es-Salaam, Tanzania; ^5^ Baylor College of Medicine, Houston, TX, United States; ^6^ Department of Oncology, Kilimanjaro Christian Medical Centre, Moshi, Kilimanjaro, Tanzania; ^7^ Catholic University of Health and Allied Sciences, Bugando, Mwanza, Tanzania; ^8^ Duke Global Health Institute, Duke University Medical Center, Durham, NC, United States

**Keywords:** cervical cancer, WLHIV, knowledge, HIV infection, awareness and Tanzania

## Abstract

**Background:**

Cervical cancer (CC) is more prevalent in women living with human immunodeficiency virus (HIV) infection compared to the general population. The magnitude is high among all countries burdened with HIV—Tanzania is no exception. Despite the unprecedented risk, women living with HIV (WLHIV) may not be aware of the risk and might have unfounded beliefs thereof. This study aimed to determine the knowledge, awareness, and beliefs on CC screening among WLHIV attending a clinic at the Kilimanjaro Christian Medical Centre (KCMC) in Northern Tanzania.

**Methods:**

This hospital-based cross-sectional study was conducted among 327 WLHIV attending care and treatment clinic (CTC) at KCMC. A pre-tested questionnaire was used to collect quantitative data. Both descriptive and regression methods were used to determine CC knowledge, awareness, and beliefs as well as factors associated with knowledge of CC among WLHIV using SPSS version 23.

**Results:**

Participants’ mean age was 46 ± 10.4 years. Although just half (54.7%) of WLHIV had insufficient knowledge of CC, the majority of the participants (83.5%) were able to recognize at least three risk factors, but with limited understanding of symptoms and prevention. The majority held positive beliefs on CC and screening practices. Factors associated with good knowledge of CC included being married (AOR: 3.66, 95% CI: 1.84–7.28), having used ART for at least 2 years (AOR: 4.08, 95% CI: 1.36–12.21), and having previously screened for CC (AOR: 1.62, 95% CI: 1.01–2.59).

**Conclusion:**

WLHIV attending care and treatment center had insufficient knowledge about CC screening. To further improve screening and treatment for CC, at both facility and community levels, targeted awareness and education campaigns are warranted.

## Introduction

Cervical cancer (CC) is the fourth most common cause of cancer-related deaths with over 300,000 deaths per year worldwide (1, 2). Approximately 85% of CC deaths occur in low- and middle-income countries (LMICs). It is the most diagnosed cancer among Tanzanian women, and the leading cause of cancer-related morbidity and mortality among women in sub-Saharan Africa (SSA) and in Tanzania (3) (4). The annual incidence of CC is 9,770 cases per 100,000 women with a mortality rate of 6,695 (5). Early treatment of precancerous lesions may reduce the incidence by an estimated 80% (2). Limited access to prevention and treatment programs increases the prevalence of advanced disease compared to high-income countries where primary and secondary prevention programs aid early detection and increase survival rates (2). Increasing CC screening coverage has been reported to reduce the CC incidence.

Women and girls contribute to 53% of 38 million people living with human immunodeficiency virus globally (6). The burden varies from one region and country to another, with more than two-thirds of the global burden of new HIV infections in SSA (7). Women living with HIV (WLHIV) are four to five times more likely to develop invasive CC compared to their counterparts in the general population. Routine screening for pre-invasive lesions remains one of the most important public health interventions that can halt the growing burden of CC (4, 8).

Addressing the burden of CC through prevention requires a knowledgeable and well-informed population, especially those at risk. The challenges of low public awareness and overall low healthcare-seeking behavior among WLHIV need to be addressed in promoting CC prevention (9–11). In 2014 in Tanzania, the Ministry of Health (MoH) introduced an integrated screening of CC within Care and Treatment Clinics (CTC) for HIV. Under this approach, women testing positive for HIV are required to start CC screening during the initiation of antiretroviral therapy (ART) and are followed up annually.

However, hindrances towards early detection include poor knowledge, limited awareness, and wrong beliefs associated with CC screening among women (12). Beliefs that cancer is untreatable have been reported to negatively affect implementation of early detection of CC among women. The belief that pap smear is painful further contributes to poor participation in CC screening (13). Lack of knowledge among WLHIV hinders CC prevention and treatment (14, 15). The knowledge gap in late-stage presentation is among the factors behind the high mortality rate in low- and middle-income countries (LMICs) (16). The challenge is more apparent in rural than in urban areas (17, 18). Evidence is scarce regarding CC knowledge, awareness, and beliefs among WLHIV in this setting. Therefore, this study was designed to assess the level of knowledge, awareness, and beliefs of CC among HIV-infected women in Tanzania who visited the CTC at Kilimanjaro Christian Medical Centre (KCMC).

## Materials and methods

### Study design and settings

This hospital-based cross-sectional study was conducted at the CTC in KCMC, a zonal referral hospital in Northern Tanzania located in Moshi urban district. KCMC hospital is among the four zonal referral hospitals in Tanzania and receives patients from five different regions of Northern Zone, including self-referrals. The CTC provides follow-up, enrollment of HIV patients including CD4 cell count, HIV viral load testing, education, and counseling in addition to other services.

### Study population

The study population included WLHIV attending the KCMC-CTC during the study period, June to September 2020. It included WLHIV aged 18–70 years who were followed for at least 1 year from the date of their HIV diagnosis. Approximately 800 WLHIV attended CTC at KCMC during that time.

### Sample size determination and sampling technique

The sample size was obtained using the precision approach with a single proportion (19). Through this approach, of the 800 WLHIV who attended CTC during the study period, a total of 327 women consented to participate and met the inclusion criteria and thus were recruited into this study. A systematic sampling method was employed to obtain the study sample. Since not all participants attended in the specific clinic day, we started with the first client of the day and used the sampling interval of two to select the subsequent participants until the end of that clinic day. All potential participants who came to CTC for follow-up visit in every clinic day were checked for eligibility criteria at the entrance desk of which the sequential numbers were given according to the arrival order. Also, to avoid multiple enrollments of the same participants, we used stickers on participant case record.

### Variables and measurements

The dependent variables of this study were knowledge, awareness, and beliefs on CC. Knowledge about CC was measured by asking participants if they had ever heard of CC, and knowledge of its symptoms and signs, risk factors, and prevention. Knowledge of the signs and symptoms was measured using 7 items; risk factors, 10 items; and prevention, 6 items. Our pre-tested questionnaire was developed with questions adapted from a tool that was used and validated in Ethiopia (20). Each correct response was given a score of 1; otherwise, a score of 0 was given when the respondent gives a wrong response or does not know the correct response. The computation of the score was made; if the participants identified at least three risk factors, they were regarded to have good knowledge, and if they identified at least three signs or symptoms of CC, they were also deemed to have good knowledge; this was the same for prevention methods: if participants identified at least three methods for prevention of CC, they were regarded to have good knowledge. Scores below these thresholds were considered poor. The conclusive definition of this categorization was from our tool and according to other studies (20).

Participants were considered “aware” if they mentioned at least one of the known methods for CC screening. These are visual inspection with acetic acid (VIA) and pap smear; otherwise, they were considered not aware about CC. Participants unable to mention at least one of these methods was regarded as “not aware”. Beliefs on CC screening were measured by using Likert scale questions. This tool is validated and has been used in educational studies in Tanzania (21). Additionally, we assessed the participants’ source of information concerning CC by using multiple response questions.

Independent variables included social characteristics and information on HIV status and CC, such as age in years, marital status, education level, occupation, number of children, ethnicity, duration living with HIV in years, duration on ART in years, and current treatment. Age was categorized as ≤45 years and ≥45 years. Marital status was characterized as single/never married, married, and divorced/widow. The level of education was categorized into three groups: primary or no formal education for those women with no formal or primary education, secondary level for women who attend at least one class in secondary school, and university/college level for those with university education level. Participant’s occupation was categorized into two: employed and unemployed. Number of children was dichotomized as having ≤2 and having ≥3.

### Data collection

A pre-tested questionnaire was used to gather data on baseline characteristics (age, level of education, marital status, occupation, and clinical characteristics), awareness, knowledge, attitude, and beliefs of CC and CC screening. This questionnaire was prepared in English and translated to Swahili; the local language used by almost everyone in Tanzania. Prior to data collection, the research team conducted a 2-day training for data collectors to familiarize them with the data collection tools, ethics, and data collection techniques. Fourth year medical students, two diploma nurses, and one master’s degree graduate nurse participated in data collection. The tool was pre-tested among 15 women with the aim of correcting inappropriate responses. We did not have changes on the developed tool. HIV data were collected from participants’ clinic records known as CTC card no 2 (CTC2). The filled questionnaires were reviewed and cross-checked before entry into SPSS 23.0 for statistical analysis.

### Data analysis

The cleaned dataset was analyzed using descriptive and regression techniques to address the specific objectives. The descriptive analyses were carried out to describe participants. To assess the factors associated with CC knowledge among WLHIV, regression analyses using binary and multivariable logistic regression analysis were performed. The univariate analyses were used to determine the factors associated with the knowledge of CC. Variables that showed associations with the knowledge and those at *p* < 0.2 were entered into the multivariate analysis model. Age was also added into the multivariate model despite the assumption of the level of association above. A 95% confidence interval with a *p*-value of less than 0.05 was regarded as statistically significant.

## Results

### Socio-demographic and general characteristics

A total of 327 WLHIV with a mean age (SD) of 46 ± 10.4 years were enrolled in this study. The majority [212 (64.8%)] had primary or non-formal education; 140 (44.6%) were divorced/widowed. More than half were unemployed [280 (85.6%)], with 47 (14.4) employed. The median number of children was 2 with an interquartile range of 2–3 children. The participants had a median duration of 12 (7–14, 22) years since being confirmed HIV positive with the majority [200 (61.2%)] having had at least 10 years since being confirmed HIV positive. The median duration on ART use was 9 (5–12, 22) years, with most of them [286 (87.5%)] being women still on first-line treatment (**Table 1**).

**Table 1:** Characteristics of the participants (*N* = 327).

Variables	n	%
Age in years		
[Mean; SD]	[46; 10.4]	
≤45	156	47.7
>45	171	52.3
Marital status		
Single, never married	62	19.0
Married	119	36.4
Divorced/widow	146	44.6
Education		
Primary/below	212	64.8
Secondary	84	25.7
College/university	31	9.5
Occupation		
Employed	47	14.4
Unemployed	280	85.6
Number of children		
[Median; IQR]	[2; 2–3]	
≤2	194	59.3
≥3	133	40.7
Ethnicity		
Chagga	206	63.0
Other	121	37.0
Duration living with HIV (years)		
[Median; IQR]	[12; 7–15]	
<5	47	14.4
5–9	80	24.4
≥10	200	61.2
Duration on ART		
[Median; IQR]	[9; 5–13]	
<2 years	24	7.4
≥2 years	300	92.6
Missing	3	0.9
Current HIV regiment		
First line	286	87.5
Second line	39	11.9
Missing	2	0.6

### Knowledge and awareness of CC among WLHIV

Regarding specific knowledge of CC, the majority of women (273; 83.5%) recognized at least three risk factors for CC. The major risk factors of CC reported by the participants included being HIV infected, low body immunity, early sexual practices, and multiple sexual partners. However, 75 (22.9%) women did not recognize that HIV-infected women are at risk of having CC, whereas many of them [182 (55.7%)] reported the use of oral contraceptives as increasing the risk of CC (**Table 2**). With regard to knowledge of symptoms and signs of CC, 191 (58.4%) women were able to mention some clinical symptoms of CC. These included unusual bleeding and pain after sexual intercourse, postmenopausal excessive vaginal bleeding, abnormal bleeding between periods, and other signs like swelling of the vagina and severe abdominal pain (**Table 2**).

**Table 2:** Knowledge and awareness of CC among WLHIV.

Items	n	%
Information about CC		
Have you ever heard about CC?	325	99.4
Can CC be associated with an infection?	187	57.2
Knowledge of risk of CC		
Mentioned at least 3 risk factors of CC	273	83.5
Multiple sexual partners within 12 months	201	61.5
Having many children ≥ 3	66	20.2
Early sexual practices before 17 years	212	64.8
Having a weakened immunity	263	80.4
Having a history of STI	239	73.1
Use of oral contraceptive pills	182	55.7
Smoking cigarette	166	50.8
Infection with human papilloma virus	52	15.9
Not using condom during sex	169	51.7
Family history of CC	106	32.4
HIV-infected women are at double risk of having cervical cancer	252	77.1
Knowledge of symptoms of CC		
**Can you name some clinical signs and symptoms of CC?**	191	58.4
Bleeding and pain after sexual intercourse	163	49.8
Post-menopausal bleeding	163	49.8
Excessive vaginal discharge	156	47.7
Abnormal bleeding between periods	158	48.3
Other signs and symptoms (e.g., swelling, severe abdominal pain)	26	8.0
Knowledge of prevention of CC		
Do you think that CC preventable?	Yes 288	88.1
By which method can cervical cancer be prevented?		
Regular medical checkup/screening	297	90.8
Vaccine for HPV	151	46.2
Delaying first sexual intercourse	190	58.1
Being faithful to one sexual partner	198	60.6
Consistent condom use	162	49.5
Have you heard about HPV vaccine?	Yes 139	42.5
Can CC be cured at early stage?	Yes 267	81.7
Awareness of CC screening methods		
Have you ever heard about CC screening?	Yes 314	96
Have you ever heard about VIA screening method?	Yes 167	51.1
Have you ever heard about pap smear screening method?	Yes 50	15.3
Should women have pap smears at least every 3 years?	Yes 3	0.9

A majority of WLHIV [288 (88.1%)] agreed that CC is preventable; regular medical checkup/screening, vaccines, and delaying first sexual intercourse were the most identified preventive measures. Ninety-six percent of the participants reported previously hearing about CC screening; however, their knowledge of CC screening methods was poor. About 51.1% were able to mention the VIA method and only 15.3% mentioned the pap smear method (**Table 2**).

In addition, most (90.8%) of the participants reported having heard information about CC from healthcare providers (doctor or nurses), followed by information from radio/television (72.7%) and relatives/friends (54.3%). Other sources of information on CC were flyers, magazines/newspaper, at work, church, and social media (such as WhatsApp, Twitter, and Instagram).

### Beliefs of CC and its screening services among WLHIV

Regarding the cause of CC, 40 (12.2%) indicated that they agreed CC is caused by the HIV virus. When it came to screening for CC, using Likert scale mean scores, 195 (59.6%) (score of 4.5) WLHIV strongly agreed that CC screening generally gives a sense of control over the course of the disease whereas 163 (49.8%) (score 4.2) reported that it is worth doing CC screening. Furthermore, 158 (48.3%) and 97 (29.7%) agreed and strongly agreed that CC screening detects pre-cancerous cells before symptoms, respectively. In addition, 207 (63.3%) strongly agreed that CC screening helps in prevention of carcinoma of the cervix. Low scores were observed on the perception that CC screening is embarrassing, very painful, and not necessary if there are no signs and symptoms, and that they were afraid to take the cancer screening test and/or worried about being diagnosed with CC (**Table 3**).

**Table 3:** Beliefs of CC and its screening services among WLHIV (*N* = 327).

Questions	Strongly disagree	Disagree	Neutral	Agree	Strongly agree	Mean
CC is caused by a virus known as HIV	150 (45.9)	69 (21.1)	68 (20.8)	40 (12.2)	00	1.99
CC screening gives you a sense of control	1 (0.3)	1 (0.3)	19 (5.8)	111 (33.9)	195 (59.6)	4.52
HIV-infected women are at double risk of having CC	7 (2.1)	11 (3.4)	57 (17.4)	108 (33.0)	144 (44.0)	4.13
It is worth to do CC screening	6 (1.8)	22 (6.7)	31 (9.5)	105 (32.1)	163 (49.8)	4.21
Carcinoma of the cervix is highly prevalent in our country and is a leading cause of deaths among all malignancies in Tanzania	3 (0.9)	10 (3.1)	45 (13.8)	164 (50.2)	105 (32.1)	4.09
CC screening detects pre-cancerous cells before symptoms	00	5 (1.5)	67 (20.5)	158 (48.3)	97 (29.7)	4.06
CC screening is very painful	50 (15.3)	45 (13.8)	128 (39.1)	74 (22.6)	30 (9.2)	2.97
HPV is responsible in causing CC	1 (0.3)	1 (0.3)	287 (87.8)	21 (6.4)	17 (5.2)	3.16
Vaginal bleeding after sex is the major sign for CC	3 (0.9)	6 (1.8)	161 (49.2)	55 (16.8)	102 (31.2)	3.76
It is embarrassing to have cervical screening	128 (39.1)	94 (28.7)	54 (16.5)	29 (8.9)	22 (6.7)	2.15
HIV-infected women need to be screened at least once every year	5 (1.5)	5 (1.5)	98 (30.0)	98 (30.0)	121 (37.0)	3.99
The screening is not necessary if there are no signs and symptoms	214 (65.4)	75 (22.9)	21 (6.4)	15 (4.6)	2 (0.6)	1.52
I am afraid to take a cancer screening test	130 (39.8)	95 (29.1)	14 (4.3)	62 (19.0)	26 (8.0)	2.26
I will be worried if I have early signs and symptoms of CC	34 (10.4)	127 (38.8)	14 (4.3)	116 (35.5)	36 (11.0)	2.98
It is difficult to go for CC screening	95 (29.1)	121 (37.0)	27 (8.3)	68 (20.8)	16 (4.9)	2.35
Screening helps in the prevention of carcinoma of the cervix	1 (0.3)	8 (2.4)	17 (5.2)	94 (28.7)	207 (63.3)	4.52

### Factors associated with knowledge of CC among WLHIV

Following bivariate analysis, variables that were significantly associated with knowledge of CC included married women (COR: 3.26, 95% CI: 1.64–6.46) as compared to unmarried or single; duration on ART of 2 years or more (COR: 3.37, 95% CI: 1.21–9.37); and ever being screened for CC (COR: 1.81, 95% CI: 1.15–2.85). After adjusting for covariates and confounders in multivariate analysis, married women were almost four times more likely to have knowledge of CC compared to their counterparts who were not married (AOR: 3.66, 95% CI: 1.84–7.28). Those who were widowed/divorced were twice more likely to have knowledge of CC compared to single women (AOR: 1.89, 95% CI: 0.95–3.75). Women who had a long duration on ART (more than 2 years) were four times more likely to understand CC compared to those with a shorter duration (less than 2 years) on ART (AOR: 4.08, 95% CI: 1.36–12.21). Furthermore, women who had ever screened for CC were almost two times more likely to be knowledgeable on CC compared to WLHIV who had never been screened for CC (AOR: 1.6295% CI: 1.01–2.59) (**Table 4**).

**Table 4:** Factors associated with knowledge of CC among women living with HIV (*N* = 327).

Variables	*N*	Knowledge of CC		Bivariate	Multivariate	*p*-value
		Poor *n* = 182	Good *n* = 148	COR (95% CI)	AOR (95% CI)	
Age in years						
≤45	156	86 (55.1)	70 (44.9)	ref		
>45	171	93 (54.4)	78 (45.6)	1.03 (0.67–1.59)	1.05 (0.65–1.69)	0.857
Marital status						
Single, never married	62	44 (71.0)	18 (29.0)	ref		
Married	119	51 (42.9)	68 (57.1)	3.26 (1.64–6.46)	3.66 (1.84–7.28)	<0.001
Divorced/widow	146	84 (57.5)	62 (42.5)	1.80 (0.95–3.44)	1.89 (0.95–3.75)	0.068
Education						
Primary/below	212	117 (55.2)	95 (44.8)	ref		
Secondary	84	49 (58.3)	35 (41.7)	0.88 (0.53–1.47)	0.83 (0.48–1.46)	0.529
College/university	31	13 (41.9)	18 (58.1)	1.71 (0.79–3.67)	1.82 (0.74–4.46)	0.191
Occupation						
Employed	47	21 (44.7)	26 (55.3)	ref		
Unemployed	280	158 (56.4)	122 (43.6)	0.62 (0.33–1.16)	0.64 (0.31–1.33)	0.227
Number of children						
≤2	194	104 (53.6)	90 (46.4)	ref		
≥3	133	75 (56.4)	58 (43.6)	0.89 (0.57–1.39)	0.71 (0.42–1.21)	
Duration living with HIV (years)						
<5	47	26 (55.3)	21 (44.7)	ref		
5-9	80	48 (60.0)	32 (40.0)	0.83 (0.40–1.72)	0.41 (0.16–1.05)	
≥10	200	105 (52.5)	95 (47.5)	1.12 (0.59–2.12)	0.46 (0.19–1.10)	
Duration on ART (*n* = 324)						
<2 years	24	19 (79.2)	5 (20.8)	ref		
≥2 years	300	159 (53.0)	141 (47.0)	3.37 (1.21–9.37)	4.08 (1.36–12.21)	0.012
Current HIV regiment (*n* = 325)						
First line	286	156 (54.5)	130 (45.5)	ref		
Second line	39	21 (53.8)	18 (46.2)	1.03 (0.53–2.01)		
Ever screened for CC						
No	143	90 (62.9)	53 (37.1)	ref		
Yes	184	89 (48.4)	95 (51.6)	1.81 (1.15–2.85)	1.62 (1.01–2.59)	0.046

COR, crude odds ratio; AOR, adjusted odds ratio.

## Discussion

CC screening information is vital to facilitate the procedure and women’s willingness to screen (23). CC screening in Tanzania includes visual inspection with acetic acid (VIA), pap smear, and, for some women, an HPV test. Both pap smear and HPV tests use cells taken from the cervix. Participants from the current study had a limited knowledge on pap smear and HPV test while few were aware of VIA. This is different from the study done by Fatima Ahmed AL-Hammad et al., which shows that 85% of participants were aware of the pap smear (24) and from the study conducted on Saudi Arabian women (25).

Knowledge of CC is important in reducing the risks, and increases the ability to prevent and control CC. Having adequate knowledge of CC was reported to increase the utilization of CC screening services among WLHIV (26). We found that 8 out of 10 women could name at least three risk factors for CC and knew it was avoidable, but they had little knowledge of symptoms and screening options. Similar findings reported the deficiency of knowledge about CC in the general population (27) with additional findings in Ethiopia (20). The inadequate grasp of CC symptoms and prevention in this study may be related to a lack of awareness among study participants, especially about the natural history of CC and the belief of CC screening. Understanding CC risk factors, causes, and prevention is crucial for women to make behavior changes.

WLHIV in this study had limited information on CC and its screening methods. The other study backs this up by reporting that WLHIV had trouble recognizing the early signs of CC (28). Similar to Shiferaw et al., who found limited understanding of CC screening methods among WLHIV, it noted that majority of participants were not able to correctly determine when women should seek care for CC screening (29). Similar findings were reported by Faustini Kimondo et al. (30). Wanyenze from Uganda identified that about half of WLHIV had limited awareness of CC screening (31). Lacking awareness of CC screening methods among WLHIV might be a result of unfriendly protocols used to educate women during their appointment visits at CTCs. This implies that WLHIV are merely informed to go for CC screening without proper information regarding CC and HIV-related risks, which causes some to have misconceptions and underlines the value of CC screening. In the research carried out by Ghufran Jassim, the difference was noticed where most of the participants were able to recognize CC and the screening methods particularly VIA and pap smear (32).

Regarding beliefs of CC screening, the majority of WLHIV felt that CC screening gives a sense of control, that it is worth doing, that it finds pre-cancerous cells before symptoms, and that it prevents cervix cancer. However, according to several women, it was reported that CC screening is uncomfortable, painful, and unnecessary if there are no symptoms. Some were afraid of a CC screening for fear of early indications and symptoms; it is hard to go. The results from the current study resemble those reported in a study from Kenya, which reported that negative beliefs of CC screening are high among WLHIV (33). These results indicate the importance of proper interventions to improve the screening technology, and it seems that the current technology, particularly the VIA method, is not user-friendly and is less appealing to the majority of women.

Married women have better CC knowledge than single ones. This can be explained by the probability of sexually transmitted signs and symptoms that cause women to seek medical attention. We suspect that married women shared CC knowledge with their spouses, which facilitate the follow up. This is different from the study conducted in Bangladesh that revealed that unmarried women were more knowledgeable on CC compared to their counterparts (34).

Having more than 2 years of ART was associated with better CC knowledge. This implies that long-time CTC attendees have heard CC information multiple times. This differs from a study conducted in Gurage zone, Southern Ethiopia, which found that individuals with plans to screen for CC and those with a family history of CC knew more about CC (35). This implies that knowledge of CC and screening procedures varies depending on various factors, and no single element explains the difference. We conducted a quantitative study, and the results do not convince us that there are other, more important aspects to consider than training and discouraging negative beliefs and misperceptions about CC screening in order to achieve the intended outcomes. For future interventions, a qualitative study could provide in-depth examinations of CC and the screening repercussions among WLHIV.

## Strengths and limitations

To our knowledge, this is the first study to explore knowledge, awareness, and beliefs on CC screening among WLHIV in this setting. Our findings have some limitations that should be taken into consideration when interpreting the results. This study was done in a referral hospital setting; therefore, the results might not resemble those of WLHIV who do not have access to a hospital. Furthermore, this study was completed at one site; therefore, results cannot be generalized to all HIV care and treatment centers in Tanzania.

## Conclusion

There was a lack of CC knowledge among HIV-positive women receiving treatment at the study facility in Northern Tanzania (KCMC). While some individuals acknowledged being aware of the HPV vaccine as a means of avoiding CC, the majority were unable to name any of the clinical signs or symptoms associated with the CC. Only half of them were even aware that VIA is used in CC screening, indicating a widespread lack of knowledge about the topic. Positive attitudes toward CC and its screening services were displayed by the participants. Knowledge of CC was observed to increase with marital status, length of time on ART, and history of CC screening.

Posters on CTC clinic walls and fliers to WLHIV could increase CC knowledge. Using the ongoing Northern zone prevention effort to educate this specific group by empowering CTC HCPs to link CC and CTC services throughout care. Increase WLHIV awareness and screening. Future studies should examine the challenges to CC screening from the healthcare provider’s perspective using qualitative approaches.

## Data availability statement

The raw data supporting the conclusions of this article will be made available by the authors, without undue reservation.

## Ethics statement

The ethical approval was sought from Kilimanjaro Christian Medical University College research ethics committee prior to the commencement of this study. This was followed by permission from the KCMC authority to use the CTC clinic for study enrollment. The patients/participants provided their written informed consent to participate in this study.

## Author contributions

DM: Conceived the research idea, designed the study, performed data analysis, prepared the manuscript and subsequent revisions. BTS, DT, and RS: Involved in data collection. EN: Participated in data entry management and manipulation for analysis. JA: Performed statistical analysis, and reviewed and edited the manuscript. JN, BCS, AM, MA, RM, EB, BS, SM, AKM, EL, and JB: Critically read and commented on the manuscript before submission to the journal. BM: Conceived the idea for the study and gave advice in designing the study, analysis, and revision of the manuscript. All authors contributed to the article and approved the submitted version.
